# Evaluation of basal rate infusion in intravenous patient-controlled analgesia for post-cesarean section pain management: A randomized pilot study

**DOI:** 10.1097/MD.0000000000037122

**Published:** 2024-02-23

**Authors:** Mi Roung Jun, Jae-Myung Kim, Jeong Yeon Kim, Ji Hoon Lee, Chae Eun Kim, Moon Ok Lee

**Affiliations:** a Department of Anesthesiology and Pain Medicine, Gyeongsang National University Hospital, Gyeongsang National University College of Medicine, Jinju, Korea; b Department of Surgery, Gyeongsang National University Hospital, Gyeongsang National University College of Medicine, Jinju, Korea; c Department of Anesthesiology and Pain Medicine, Samsung Changwon Hospital, Sungkyunkwan University School of Medicine, Changwon, Korea.

**Keywords:** analgesia, cesarean section, fentanyl, patient-controlled analgesia

## Abstract

**Objective:**

Administering opioids via intravenous patient-controlled analgesia is a prevalent approach for managing postoperative pain. Nevertheless, due to concerns about opioid-related side effects and the potential for opioid tolerance, there is a growing emphasis on adopting opioid-sparing techniques for postoperative pain management. We aimed to investigate the effect of adding a basal rate infusion in fentanyl-based IVA following a cesarean section (CS).

**Method:**

Forty-eight patients, who received pain management through IVA after CS, were assigned randomly into 3 groups based on the background rate setting: Group 0 (0 mcg/hour, n = 16), Group 1 (15 mcg/hour, n = 16), and Group 2 (30 mcg/hour, n = 16). We assessed the impact of the basal infusion rate on opioid consumption and the visual analog scale (VAS) scores during the first 48 hours post-CS and also investigated opioid-induced side effects and the requirement for rescue analgesics in the ward during the first 48 hours after CS.

**Results:**

In the initial 24 hours following CS, fentanyl consumption significantly increased in Group 2 compared with Group 0 and Group 1 (*P* = .037). At 24 hours, VAS scores both at rest and during movement, tended to decrease, as the basal rate increased; however, no significant differences were observed between the groups (*P* = .218 and 0.827, respectively). Between the first 24- and 48-hours post-CS, fentanyl consumption showed a marked increase in both Group 1 and Group 2 compared to Group 0 (*P* < .001). At 48 hours, the VAS scores at rest displayed a trend toward reduction; however, no significant differences between groups were evident (*P *= .165). Although the incidence of opioid-induced complications was noted, no statistically significant differences were recorded between groups during the initial 24 hours and subsequent 24 to 48 hours period (*P* = .556 and *P* = .345, respectively).

**Conclusion:**

The inclusion of a basal fentanyl infusion in the IVA protocol did not provide any advantages over an IVA devoid of a basal rate infusion in managing acute pain following CS.

## 1. Introduction

Achieving optimal pain management after a cesarean section (CS) is essential to ensure that the mother can effectively care for her baby. However, it is equally important to tailor the treatment plan to minimize any potential effects on breastfeeding and mitigate maternal complications related to analgesic use. Therefore, the guideline for pain management following CS recommended anesthetic and analgesic approaches throughout pre-, intra-, and postoperative periods, also including considerations for surgical techniques.^[1]^ In this study, our emphasis was on the analgesic aspect for pain control during the postoperative period after CS.

The administration of opioids via intravenous patient-controlled analgesia (IV PCA; IVA) is a common practice for the control of postoperative pain.^[2]^ Among opioids employed in IVA, fentanyl has been shown to have a lower risk of nausea, vomiting, pruritis, and respiratory depression with higher patient satisfaction.^[3]^ Fentanyl, being a short-acting opioid with a rapid onset, is considered suitable as an on-demand dose in IVA. However, due to its rapid offset, the use of a basal dose has been proposed to ensure adequate pain relief.^[4]^ Moreover, the implementation of an infusion device programmed for fentanyl PCA, which automatically adjusts the baseline infusion dose based on the individual patient’s requirements, has demonstrated greater efficacy than a constant rate background infusion in postoperative pain management.^[5]^

Nevertheless, in the context of enhanced recovery after surgery (ERAS) and driven by concerns regarding opioid-related side effects and opioid tolerance,^[6]^ there is a growing emphasis on opioid-sparing or even opioid-free techniques for postoperative pain management. Based on the current situation, we endeavored to identify the necessity of a basal infusion rate in fentanyl-based IVA for reducing overall opioid consumption for controlling acute pain after CS.

## 2. Methods

### 2.1. Patient population and postoperative analgesia

This study was approved by the institutional review board of our hospital (Ref. SCMC 2022-10-009) and registered with the International Clinical Trials Registry Platform (http://cris.nih.go.kr). Written informed consent was obtained from 48 patients scheduled for elective CS who were prescribed intravenous patient-controlled analgesia (IV PCA; IVA) between November 2022 and April 2023. The patients eligible for the study were aged 18 years or older with American Society of Anesthesiologists physical status 1 or 2. Patients with allergies to opioids, non-opioid analgesics, and local anesthetics; those with a history of drug abuse; or those without continuous wound infusion catheter, undergoing emergency CS, and patients who stopped IVA due to side effects were excluded.

The administration protocol for IVA was as follows:

- Prepare a mixture of fentanyl 2250 mcg, nefopam 60 mg, paracetamol 3 g, and ramosetron 0.6 mg in 150 mL of normal saline solution.
- Set the background rate at 0, 15, or 30 mcg/hour (0, 1, 2 mL), the bolus dose at 30 mcg (2 mL), and the lockout time at 10 minutes.

Based on the background rate setting, randomly assign the patients into 3 groups using a computer-generated randomization list:

- Group 0 (basal rate 0 mcg/hour (0 mL), n = 16)
- Group 1 (basal rate 15 mcg/hour (1 mL), n = 16)
- Group 2 (basal rate 30 mcg/hour (2 mL), n = 16)

The IVA was connected to the patient at the start of skin closure during the CS procedure. If the visual analog scale (VAS) was 5 or higher at rest and the patients requested, additional rescue analgesics were administered.

The analgesic plan implemented by the obstetric team is as follows:

All patients had an in-wound catheter inserted beneath the fascia, which was connected to a pump (AutoFuser® AceMedical Corp., Ltd., Korea) containing 0.6% ropivacaine 100 mL, running at a rate of 3 to 4 mL/h for 24 hours. Additionally, a scheduled administration of 750 mg of intravenous acetaminophen was provided to all patients upon their arrival at the ward and in the morning until postoperative day 2.

The primary outcome of this study was to investigate the effects of the basal infusion rate on opioid consumption and the VAS scores during the 48 hours following CS. Secondary outcomes included assessing opioid-induced side effects and the need for rescue analgesics in the ward based on the background infusion rate during the 48 hours after CS. To this end, we visited patients at 24- and 48-hours postsurgery to collect relevant data. VAS scores were divided into pain at rest, and during movement such as sitting, coughing, or walking. The overall opioid-related side effects were defined as the presence of at least one of the following 3 side effects: nausea, vomiting, dizziness, urinary retention, sedation and respiratory depression.

### 2.2. Sample size

When estimating the sample size for the pilot trial, Julious study recommended a minimum sample size of 12 subjects per treatment arm.^[7]^ In this study, a 30% dropout rate was applied to account for potential attrition. As a result, the final sample size was determined to be 16 subjects per group.

### 2.3. Statistical analysis

A one-way analysis of variance or the Kruskal–Wallis test, followed by multiple comparison procedures using Duncan method, was conducted to compare the 3 groups. Categorical variables were compared between groups using Pearson chi-square test or Fisher exact test. Continuous variables were presented as medians (25–75th percentile) or means ± standard deviation, while categorical variables were presented as percentages. All statistical analyses were performed using SigmaPlot 14.5 for Windows (Systat Software, Inc., Chicago, IL) and Stata 15.1 (Stata Corporation, College Station, TX). The level of statistical significance was set at a *P* value <.05.

## 3. Results

A total of 48 patients was eligible for the study. There were no patients who met the exclusion criteria. Patient characteristics that showed no significant differences between groups are summarized in Table 1. In our study, there were no serious complications related to opioids such as sedation and respiratory depression. The main opioid-related side effects observed were nausea and dizziness.

**Table 1:** Patients’ characteristics.

	Group 0 (n = 16)	Group 1 (n = 16)	Group 2 (n = 16)	*P*
Age (year)	32.94 ± 3.07	32.88 ± 3.95	33.75 ± 5.22	.804
Height (cm)	165.00 (158.25–167.00)	164.00 (157.00–167.00)	164.50 (161.25–166.75)	.825
Weight (kg)	75.05 (70.00–79.40)	68.50 (62.00–78.00)	72.65 (69.25–79.50)	.423
Body mass index (kg/m^2^)	29.00 (24.59–31.66)	27.14 (23.12–30.93)	27.69 (25.68–29.19)	.531
Anesthesia General Spinal	1 (6.25) 15 (93.75)	5 (31.25) 11 (68.75)	5 (31.25) 11 (68.75)	.176

Values expressed in median (25th–75th percentiles), means ± standard deviation, or number (%).

*There is a significant difference versus Group 0 (*P* < .05).

†There is a significant difference versus Group 1 (*P* < .05).

**Figure 1.**
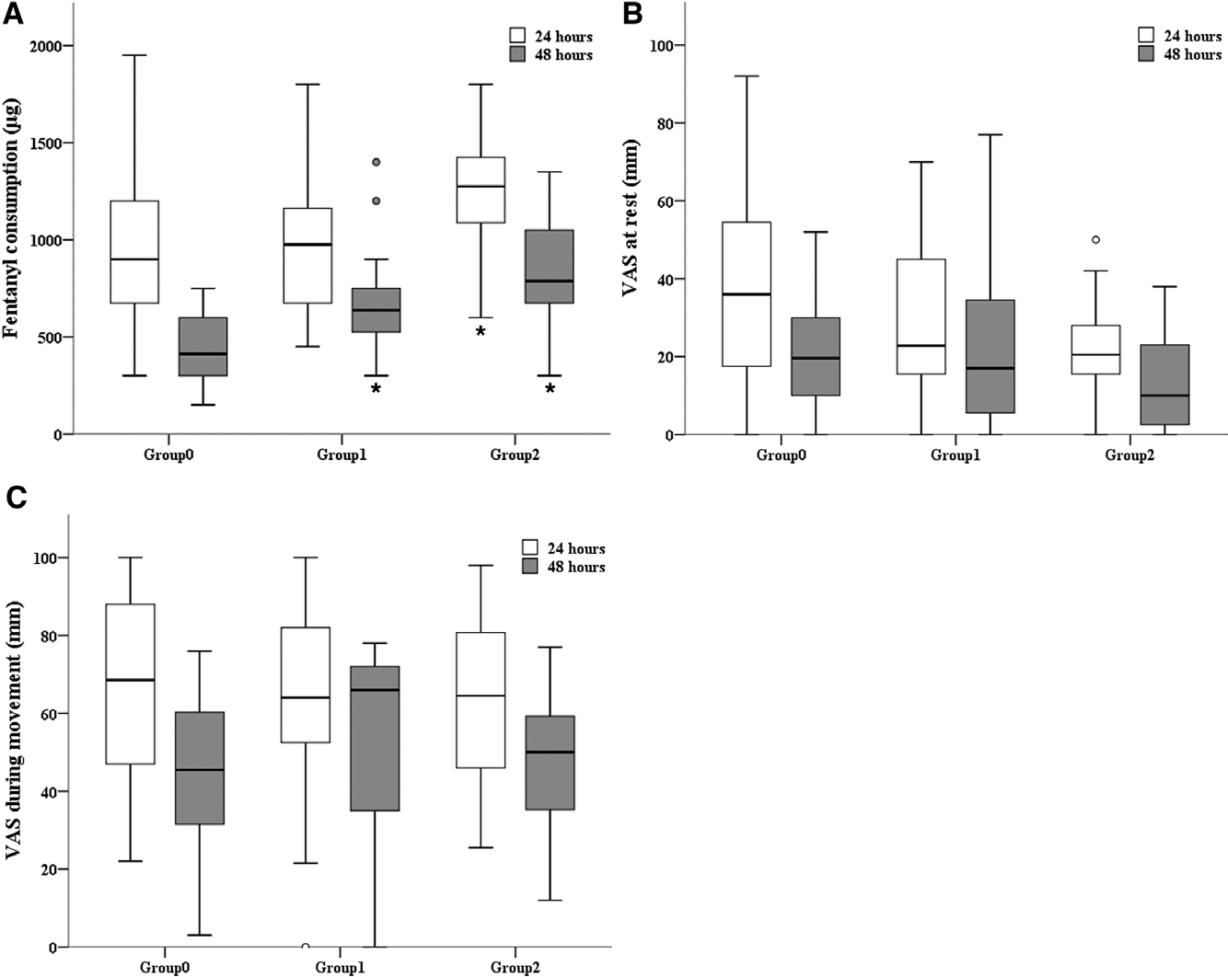
Comparison of fentanyl consumption (A) via intravenous patient-controlled analgesia (IV PCA; IVA), and 100 mm visual analogue scale (VAS) scores at rest (B) and during movement (C) within the first postoperative 24 and 48 hours (h) among 3 groups. *There is a significant difference vs Group 0 (*P* < .05). †There is a significant difference vs Group 1 (*P* < .05).

During the first 24 hours after CS, fentanyl consumption was significantly increased in Group 2 compared with Group 0 and Group 1 (*P* = .037, Fig. 1 and Table 2). At 24 hours, the VAS scores both at rest and during movement tended to decrease, while the basal rate increased; however, there were no significant differences between groups (*P* = .218 and 0.827, respectively, Fig. 1 and Table 2). Although opioid-induced complications such as nausea and dizziness occurred, there were no statistically significant differences between groups (*P* = .556, Table 2). The rescue analgesic, pethidine 50 mg, was prescribed only to one patient in Group 1 during the first 24 hours after CS (Table 2). No patients required additional analgesics in Group 0 and Group 2.

**Table 2:** Comparison of fentanyl consumption and side effects of intravenous patient-controlled analgesia (IV PCA; IVA), 100 mm visual analogue scale (VAS) scores and rescue analgesics in the ward within postoperative 24 hours and 48 hours among 3 groups.

	Group C (n = 16)	Group 1 (n = 16)	Group 2 (n = 16)	*P*
During the first 24 hours				
Fentanyl consumption (mcg)	956.25 ± 427.35	979.69 ± 369.94	1270.31 ± 306.76*,†	.037
At 24 hours VAS at rest (mm)	36.00 (15.75–56.75)	24.90 (16.50–47.25)	20.50 (14.50–31.50)	.218
At 24 hours VAS during movement (mm)	66.28 ± 22.93	65.31 ± 21.62	61.72 ± 21.54	.827
Opioid-induced side effects				.556
Yes	3 (18.75)	5 (31.25)	2 (12.50)	
Nausea	2	1	2	
Dizziness	2	4	1	
No	13 (81.25)	11 (68.75)	13 (87.50)	
Rescue analgesics				.098
Pethidine (n)	0	1	0	
Dose (mg)		50		
During the first 24 to 48 hours				
Fentanyl consumption (mcg)	445.31 ± 200.25	696.88 ± 280.01*	810.94 ± 272.79*	<.001
At 48 hours VAS at rest (mm)	19.55 (9.50–31.00)	17.95 (7.13–36.38)-	10.00 (1.25–25.50)	.165
At 48 hours VAS during movement (mm)	46.63 ± 19.90	56.55 ± 20.85	47.22 ± 17.10	.198
Opioid-induced side effects				.345
Yes	0 (0.00)	2 (12.50)	3 (18.75)	
Nausea	0	1	3	
Dizziness	0	1	1	
No	16 (100.00)	14 (87.50)	13 (81.25)	
Rescue analgesics Pethidine (n) Dose (mg)	0	0	0	.452

Values expressed in median (25th–75th percentiles), means ± standard deviation, or number (%).

* There is significant difference versus Group C (*P* < .05).

† There is significant difference versus Group 1 (*P* < .05).

During the first 24 to 48 hours after CS, fentanyl consumption was significantly increased in Group 1 and Group 2 compared with Group 0 (*P* < .001, Fig. 1 and Table 2). At 48 hours, the VAS scores at rest tended to decline; however, there were no significant differences between groups (*P *= .165, Fig. 1 and Table 2). On the other hand, the VAS scores during movement were higher in Groups 1 and 2 than in Group 0, but there were no statistically significant differences between groups (*P *= .198, Fig. 1 and Table 2). While the occurrence of opioid-induced complications, such as nausea and dizziness, was more frequent in Groups 1 and 2, no statistically significant differenced were observed between the groups (*P* = .345, Table 2). The rescue analgesic was not prescribed during the first 24 to 48 hours after CS in all groups (Table 2).

The cumulative fentanyl consumption over the first 48-hour period after CS was significantly higher in Group 2 in comparison to Groups 0 and 1 (*P *= .001, Table 3).

**Table 3:** Comparison of total fentanyl consumption among 3 groups over the first 48 hours post-cesarean section (CS).

	Group 0 (n = 16)	Group 1 (n = 16)	Group 2 (n = 16)	*P*
Total fentanyl consumption	1312.50 (975.00– 1781.00)	1650.00 (1200.00– 2243.75)	2250.00*,† (1987.50–2250.00)	.001

Values expressed in median (25th–75th percentiles).

* There is significant difference vs Group 0 (*P *< .05).

† There is significant difference vs Group 1 (*P *< .05).

## 4. Discussion

In this study, lowering or eliminating the basal rate of intravenous patient-controlled analgesia (IV PCA; IVA) to 15 mcg/h (Group 1) and 0 mcg/h (Group 0) resulted in a significant reduction of fentanyl consumption during the first 48 hours after CS. Despite a tendency for VAS scores to increase as the basal rate of IVA declined, there was no significant difference observed between the groups.

Managing moderate to severe acute pain following different types of surgery presents several challenges, despite the availability of pharmacological and non-pharmacological strategies.^[8]^ In a study by Gerbershagen et al, CS ranked as the ninth most painful of 179 procedures.^[9]^ Insufficient management of acute pain following CS has been linked to chronic pain, delayed surgical recovery, and postpartum depression.^[10]^

In the context of postoperative acute pain management, variable factors come into consideration, encompassing both surgical and anesthetic factors that influence pain.^[11]^ Considerations related to the surgical factors involve the type and duration of surgery.^[11,12]^ In terms of anesthetic factors, the choice between general or regional anesthesia (RA), alongside the specific type of analgesics utilized, holds the potential to influence postoperative pain.^[13]^ In our study, we deliberately directed our attention to anesthetic factors, specifically exploring the significant implications that both the chosen anesthetic techniques and the postsurgical analgesic method could have on pain management in patients following CS. The RA has been recognized as a favorable method, ensuring the safety of both maternal and fetal well-being post-CS.^[14,15]^ Beyond CS, a recent study by Corte et al documented that RA exhibited a sustained analgesic effect, providing assurance of an expedited recovery, even in laparoscopic gynecologic surgery.^[16]^ Conversely, multiple studies have indicated that RA does not demonstrate superiority concerning maternal and fetal health, as well as pain control following CS, and no significant advantages after laparoscopic gynecologic procedures.^[17,18]^ In our study, the number of patients undergoing general anesthesia in each group did not show statistically significant differences compared to those undergoing RA between the groups. However, future studies on post-CS pain control should thoroughly evaluate not only opioid consumption, opioid-related adverse effects, and complications but also the influence of the chosen anesthetic method. Consequently, we have chosen to center our study on the postoperative pain control regimen after CS. For post-CS pain relief, employment of multimodal analgesic regimens that incorporate a combination of opioids, nonsteroidal anti-inflammatory drugs, acetaminophen/paracetamol, ketamine, gabapentin and nefopam is recommended.^[19,20]^ Among them, opioids are the leading pharmacological option for mitigating moderate to severe surgical pain.^[8]^

IVA, primarily using opioids such as morphine, oxycodone, and fentanyl, is widely adopted as the standard approach for managing acute, chronic, and postoperative pain.^[21]^ According to McNicol et al, PCA is associated with higher patient satisfaction and has demonstrated effectiveness in pain management.^[21]^ Although PCA has been linked to higher opioid consumption, the occurrence of PCA-related side effects did not significantly differ compared to non-PCA groups.^[21]^ Consequently, they proposed that the use of PCA should be considered when it is deemed necessary and appropriate for analgesia.^[21]^ With IVA, patients are able to self-administer opioids using a preprogrammed infusion pump. Determination of the recommended initial loading dose, demand dose, and background infusion rate for IVA is contingent upon the specific opioid employed.^[2]^ Hence, it is imperative to ascertain suitable variables for IVA and to select appropriate drugs based on the anticipated intensity of postoperative pain.

Based on our prior study, our primary strategy for managing acute pain in all CS patients involved the use of IVA with a basal rate infusion.^[22]^ However, ongoing debates persist regarding the optimal setting for the background infusion rate in IVA. The inclusion of a basal infusion has demonstrated effectiveness in managing pain following surgery, albeit accompanied by a higher incidence of mild adverse effects.^[4,5,23]^ In contrast, numerous studies have shown that supplementing continuous infusion to IVA resulted in an overall increase in opioid consumption and side effects, without yielding any discernible benefits in pain control.^[24–26]^ In our current study, we observed a significant increase in total fentanyl consumption over a 48-hour period in Group 2 compared with Groups 0 and 1. Furthermore, fentanyl consumption during the 24 to 48 hours timeframe was significantly higher in Groups 1 and 2 compared with Group 0. These findings indicated that the continuous infusion of the established basal rate could not adjust the dosage of drug administration in response to decreasing pain levels over the course of time following surgery. This could lead to the overuse of opioids and an increased likelihood of side effects. However, there were no significant differences among the 3 groups in terms of VAS scores (at rest and during movement). Moreover, VAS scores during movement in the 48-hour period trended higher in Groups 1 and 2 compared with Group 0, but the differences were not statistically significant. These findings suggested that the use of a basal infusion may not be necessary for managing pain, especially during motion, as pain scores tended to decrease over time. Our results aligned with previous research highlighting the superior effectiveness of opioids in alleviating pain during periods of rest.^[6]^ Additionally, our study findings support the research proposed by Notcutt et al, which suggested that for surgeries expected to involve severe pain, increasing bolus doses may be effective than relying solely on a background infusion rate.^[27]^ Doubts have been raised about the necessity of basal infusion rate and even requirement of administering opioids via IVA.

The increasing concern about the potential negative effects of opioids has led to a shift toward reducing their usage in managing postoperative pain after CS. One recent approach known as ERAS in CS focused on using a combination of pain management techniques to minimize the need for opioids.^[28]^ In addition, the procedure-specific postoperative pain management guidelines for elective CS did not recommend the use of opioid-based PCA for pain management.^[1]^ For example, a study by Macias et al showed that a regimen combining opioids with scheduled non-opioid drugs significantly reduced opioid use in acute pain management after CS.^[29]^ Their study also demonstrated that a multimodal approach, without implementing other ERAS protocols, was sufficient to alleviate pain scores compared to conventional morphine-based PCA.^[29]^ In our hospital, as part of the multimodal analgesia approach, we supplement the IVA regimen with nefopam 60 mg and paracetamol 3 g. Furthermore, the obstetrics team inserts an in-wound catheter beneath the fascia, which was connected to a pump containing 0.6% ropivacaine 100 ml. Additionally, a scheduled administration of 750 mg of intravenous acetaminophen was provided to all patients upon their arrival at the ward and in the morning until postoperative day 2. Throughout the study period, only one patient required pethidine 50 mg as a rescue analgesic, indicating a reduction in the use of additional pain-relieving drugs compared with our previous study.^[22]^ On the other hand, perioperative injection of acetaminophen, ketorolac, and subcutaneous bupivacaine as a part of ERAS protocols did not lead to a decrease in opioid consumption, and clinicians tended to prescribe more opioids after CS.^[30]^ Interestingly, Teigen et al applied ketorolac to the ERAS group regularly for 24 hours after CS, but it did not reduce postoperative opioid consumption or facilitate early hospital discharge.^[31]^ Further studies are needed to evaluate the correlation between the multimodal approach and the necessity of opioids for acute pain control after CS.

This study has several limitations. First, we did not subdivide and analyze patients according to the anesthetic protocol, whether general or spinal anesthesia was used, which could potentially impact fentanyl consumption in IVA. The effect of RA on postoperative pain control remains a subject of debate. However, different anesthesia techniques may influence postoperative pain levels and, consequently, the demand for opioids through IVA, indicating a need for more in-depth investigation after CS. Second, we did not collect data about patient satisfaction, which is an important factor in assessing the success of pain control. Evaluating individual patient satisfaction would provide valuable information for tailoring pain management protocols and improving patient experiences in the post-CS setting. Third, our study did not encompass data collection on surgery-related factors, potentially influencing the study’s outcomes. Finally, our study is constrained by a small number of patients. Despite the pilot nature of our study, the limited sample size, as per the framework outlined by Julius,^[7]^ impaired the strength of our conclusions. Large-scale studies, considering patient satisfaction and implementing effective methods for controlling postoperative pain, are necessary to achieve optimal outcomes in postoperative pain management following CS. These limitations mentioned above represent potential weaknesses in our study. However, to our knowledge, there are still a limited number of comparative studies between groups without a basal rate and those with a basal rate in IVA post-CS. We anticipate that our study will lead to further research in this field.

In conclusion, the addition of a basal fentanyl infusion to IVA did not confer any advantages over IVA without a basal infusion for acute pain management after CS. On the contrary, fentanyl consumption increased in groups with a basal infusion, with no statistically significant differences observed in VAS scores. Despite the anticipated acute and intense pain following surgeries such as CS, it is advisable to implement a multimodal pain control approach independent of relying solely on opioids.

## Author contributions

**Conceptualization:** Mi Roung Jun, Jae-Myung Kim, Jeong Yeon Kim.

**Data curation:** Jeong Yeon Kim, Ji Hoon Lee, Chae Eun Kim.

**Formal analysis:** Mi Roung Jun, Moon Ok Lee, Ji Hoon Lee, Chae Eun Kim.

**Investigation:** Mi Roung Jun, Moon Ok Lee, Jeong Yeon Kim.

**Supervision:** Mi Roung Jun.

**Writing – original draft:** Mi Roung Jun, Jae-Myung Kim, Jeong Yeon Kim.

**Writing – review & editing:** Mi Roung Jun, Moon Ok Lee, Jeong Yeon Kim.
